# Multidimensional Lifestyle Profiles and Psychological Outcomes in University Students: A Cluster Analysis Approach

**DOI:** 10.17505/jpor.2026.29454

**Published:** 2026-08-24

**Authors:** Emanuela Rabaglietti, Aurelia De Lorenzo, Margherita Micheletti Cremasco, Francesco Sguaizer, Anna Mulasso, Alberto Rainoldi, Paolo Riccardo Brustio

**Affiliations:** 1 Se-Crea Research Group, Department of Psychology, University of Torino, Torino, Italy; 2 Department of Life Sciences and Systems Biology, University of Torino, Torino, Italy; 3 Neuromuscular Function Research Group, School of Exercise & Sport Sciences, University of Torino, Torino, Italy; 4 Department of Medical Sciences, University of Torino, Torino, Italy; 5 Department of Clinical and Biological Sciences, University of Torino, Torino, Italy

**Keywords:** young adult, anxiety, cluster analysis, depression, lifestyle, students, university, wellbeing

## Abstract

**Background:**

Emerging adulthood is a critical period for the adoption of health behaviours. This study aimed to identify distinct lifestyle profiles among university students using a person-oriented approach and to examine their associations with psychological wellbeing, stress, anxiety, and depression.

**Methods:**

In this cross-sectional study, 2,078 students (aged 18–39) from the University of Torino provided data on six lifestyle indicators: sedentary behaviour, diet, sleep, alcohol, sport, and smoking. Cluster analysis (Partitioning Around Medoids) was used to identify profiles. Differences in psychological outcomes (WEMWBS, PSS-10, HADS) were assessed using ANCOVA, adjusting for age and gender.

**Results:**

Four profiles were identified: Active–Healthier (29%), Sedentary–Sporty (17.6%), Sedentary–Non-sporty (25.7%), and Active–Riskier (27.7%). Sedentary behaviour showed the strongest differentiation across profiles (Cramér’s *V* = 0.789). After adjustment for age and gender, lifestyle profile membership was significantly associated with wellbeing, perceived stress, anxiety, and depressive symptoms (all *p* < .001), although effect sizes were small (partial *η*^*2*^ = 0.009–0.016). The Active–Healthier profile showed the most favourable psychological pattern, whereas differences among the remaining three profiles were not statistically significant.

**Conclusion:**

Health-related behaviours co-occur in multidimensional patterns among university students. Person-oriented profiling may help universities move beyond generic health promotion towards more targeted interventions that jointly address physical and mental health.

## Introduction

Scientific literature often identifies university students who are commonly studied as a subgroup of young adulthood, a stage characterized by significant developmental and ecological changes (Collins et al., 2023; Lucini et al., 2024; Wickham et al., 2020). This period, also known as emerging adulthood (Arnett, 2023), involves a redefinition of social roles, increased personal responsibilities, and often the transition to independent living away from the family home (Simões de Almeida et al., 2025; Worsley et al., 2021). Leaving established routines fosters greater decision-making autonomy, which is reflected in health management and the adoption of daily behaviours related to nutrition, physical activity, and stress management (Franchini et al., 2025; Lucini et al., 2024). However, this growing independence, combined with academic pressures and the need to form new social relationships, makes university students a particularly vulnerable population (Sánchez Mata & Tama Quiñones, 2025; Simões de Almeida et al., 2025). They differ from both adolescents and older adults in characteristics that influence decision-making, behaviours, and choices related to health (Lucini et al., 2024). Decisions made during university years affect not only immediate mental and physical wellbeing but also tend to establish behavioural patterns that can persist into adulthood (Doak et al., 2023; Lonati et al., 2024). For these reasons, the scientific community considers the university period a key window of opportunity for health promotion interventions and primary prevention of chronic noncommunicable diseases (NCDs), including cardiovascular disease, diabetes, and metabolic disorders (Lonati et al., 2024; Lucini et al., 2024; Wickham et al., 2020).

Despite the high preventive potential and relevance of this developmental stage, the literature highlights a marked discrepancy between international public health recommendations and the behaviours actually adopted by university students (Doak et al., 2023; Lonati et al., 2024). For example, although the World Health Organization (WHO) recommends 150–300 min/week of moderate-intensity aerobic activity or 75–150 min/week of vigorous-intensity activity, a substantial proportion of university students do not meet these recommendations (Lonati et al., 2024; Sansone et al., 2025). Moreover, sedentary behaviour is common in academic settings, with students spending several hours per day sitting on weekdays (e.g., studying or using electronic devices) (Lucini et al., 2024; Sansone et al., 2025). Physical inactivity is often associated with specific perceived barriers, including lack of time due to academic commitments and low motivation, which are among the main reasons for not maintaining an active lifestyle (Çetinkaya & Sert, 2021; Oftedal et al., 2024; Teleman et al., 2015). At the same time, dietary habits among university students often shift toward ‘Western’ patterns, characterized by higher intake of ultraprocessed foods, red meat, and added sugars (Franchini et al., 2025; Lonati et al., 2024; Wickham et al., 2020). In the scientific literature, the Mediterranean Diet (MD) is widely recognized as one of the healthiest dietary patterns for promoting health and wellbeing. It is considered a reference model due to its benefits in reducing overweight and obesity, preventing chronic diseases, and promoting longevity (Garcí a - Fernández et al., 2014; Serhan & Julien, 2024). Adherence to the MD has also been associated with a significant reduction in overall mortality, as well as lower risk of cardiovascular and neurodegenerative diseases, cancer incidence, and diabetes (Dinu et al., 2018). Importantly, these health benefits may occur independently of weight loss (Lotti et al., 2025). According to the literature, only a small proportion of students, estimated at around 3.4% in some cultural contexts, achieve the recommended daily intake of five portions of fruit and vegetables (Moscatelli et al., 2023; Sansone et al., 2025; Simões de Almeida et al., 2025). As a result, the overall quality of the diet is often classified as “low” or “intermediate” based on international nutritional standards (Franchini et al., 2025; Sansone et al., 2025). These patterns may be even less favourable among students living away from home, far from their family environment (Franchini et al., 2025; Simões de Almeida et al., 2025). This situation is also associated with poor sleep hygiene, defined as a set of inappropriate behaviours and environmental conditions that interfere with night-time rest and promote insomnia or daytime sleepiness (Simões de Almeida et al., 2025; Wickham et al., 2020). Numerous studies report irregular sleep patterns and insufficient sleep duration, conditions that compromise cognitive functioning and academic performance and increase the risk of developing mood disorders (Altun et al., 2012; Oftedal et al., 2024; Smith & Lee, 2022). Finally, the consumption of substances such as alcohol and tobacco remain high in this population (Çetinkaya & Sert, 2021; Lucini et al., 2024; Simões de Almeida et al., 2025). In particular, alcohol use is often reported as a coping strategy for academic stress and social pressures and as a facilitator of social interactions (Collins et al., 2023; Doak et al., 2023; Simões de Almeida et al., 2025).

Evidence suggests that lifestyle behaviours do not occur in isolation but tend to co-occur within multidimensional risk profiles (Lucini et al., 2024). For example, poor diet quality is frequently observed alongside higher body mass index (BMI) and cigarette smoking (Lucini et al., 2024). Similarly, several studies describe the bidirectional relationship between psychological stress and lifestyle behaviours (Hanawi et al., 2020; Simões de Almeida et al., 2025). Higher perceived stress has been associated with less healthy habits (e.g., greater intake of foods rich in fat and sugar) and with more sedentary behaviours (Hanawi et al., 2020; Lucini et al., 2024). Conversely, healthier behaviours are a protective factor for managing anxiety and depression symptoms (Simões de Almeida et al., 2025). Understanding these interconnections is particularly relevant because chronic metabolic and inflammatory conditions are often preceded by years of unhealthy behaviours, which in young adults may occur before overt clinical alterations are evident (Lucini et al., 2024). In line with the multidimensional nature of risk profiles, the literature reports a growing concern regarding the mental health of university students, with an increase in the prevalence of stress, anxiety, and depressive symptoms (Lucini et al., 2024; Simões de Almeida et al., 2025). Students are particularly vulnerable due to high performance demands and the transition to independence, often without adequate individual or social coping resources (Simões de Almeida et al., 2025). In response to academic and social pressures, many students adopt dysfunctional coping strategies, such as alcohol consumption, tobacco smoking, or increased sedentary behaviour (Lucini et al., 2024; Simões de Almeida et al., 2025). These behaviours can contribute to a vicious cycle, in which poorer lifestyle patterns co-occur with greater anxiety and depressive symptoms, with potential long-term implications for cardiometabolic health (Lucini et al., 2024). Stress has also been shown to negatively affect sleep quality and appetite, leading to reduced fruit and vegetable consumption and increased intake of energy-dense foods (Hanawi et al., 2020; Lucini et al., 2024).

However, despite evidence that that unhealthy behaviours may increase the risk of vicious cycles during university years (Lucini et al., 2024; Simões de Almeida et al., 2025), promoting behavioural change in young adults requires a different approach than traditional models that focus exclusively on preventing future clinical risks (Collins et al., 2023; Wickham et al., 2020). In this age group, clinical indicators such as blood pressure and cholesterol levels are frequently within normal limits (Trejo et al., 2018). As a result, many young people perceive themselves as healthy and may be less motivated to prevent diseases that only manifest in the long term (Lucini et al., 2024). For this reason, communication strategies are more effective when they emphasize the immediate benefits of an active lifestyle and a balanced diet, highlighting improvements in subjective wellbeing, vitality, and present quality of life (Oftedal et al., 2024; Wickham et al., 2020). At the same time, it is important to engage young adults in preventive services and health campaigns because these services are often perceived as being intended exclusively for people with established or chronic conditions (Luquis & Kensinger, 2017). In this context, early identification of co-occurring lifestyle patterns (i.e., clusters of unhealthy behaviours), before the onset of obvious clinical changes, is a fundamental step in promoting lasting and sustainable changes in students’ daily routines through tailored interventions (Collins et al., 2023; Luquis & Kensinger, 2017).

In this context, academic institutions no longer perform an exclusively educational function but can be considered true public health hubs, playing a significant role in shaping the wellbeing trajectories of young adults (de Lima et al., 2025; Doak et al., 2023). Across several European contexts, universities are increasingly implementing initiatives to promote healthier and more sustainable lifestyles and to improve quality of life within academic communities (Franchini et al., 2025; Lucini et al., 2024). However, the literature indicates that generic interventions and standardized programs are often limited in their effectiveness at producing significant behavioural change (Lucini et al., 2024; Paduano et al., 2025). One contributing factor is the reliance on variable-oriented approaches that examine single risk factors in isolation, potentially overlooking how behaviours cluster within individuals (Collins et al., 2023; Lucini et al., 2024; Simões de Almeida et al., 2025). To address these limitations, a paradigm shift toward a person-oriented approach is needed (Collins et al., 2023; Lucini et al., 2024; Smith & Lee, 2022). This approach enables mapping the student population using cluster analysis techniques and identifying multidimensional risk profiles based on combinations of multiple behaviours (Bennasar-Veny et al., 2020; van Hooijdonk et al., 2023; Zurc & Majerič, 2025). Identifying these subgroups can inform tailored strategies and targeted educational interventions, potentially making campus initiatives more responsive to the needs of higher-risk students (Carvalho & Vilaça, 2024; Doak et al., 2023; Oftedal et al., 2024). These profiles are not merely descriptive but represent operational configurations useful for planning and implementing effective prevention interventions (Lucini et al., 2024).

However, although person-oriented studies have begun to describe lifestyle profiles in university populations, evidence remains limited on whether concise multi-behaviour profiles (built from indicators readily measurable in campus surveys) are systematically associated with key psychological outcomes. This limits the ability of universities to design integrated, targeted interventions addressing both lifestyle and mental-health dimensions within the same student subgroups. Based on this rationale, the primary aim of this cross-sectional study was to identify distinct lifestyle profiles among university students, using a person-oriented cluster analysis approach, with indicators selected for feasibility and potential use in campus health planning. We hypothesized that less healthy profiles would show worse perceived wellbeing and higher stress, anxiety, and depressive symptoms.

## Materials and Methods

### Study design and participants

This cross-sectional study was conducted among students enrolled at the University of Torino (UniTO; https://www.unito.it) within the Wellness for Students (W4S) project. The W4S project is the result of collaboration among various departments at the University of Torino, promoting well-being among university students in psychological, anthropometric, physical, and biological areas. Data were collected between October 2023 to May 2025. Students were invited via an institutional email introducing the survey and providing a direct link to the questionnaire. The survey was administered anonymously via LimeSurvey. Data were collected anonymously and only participants who fully completed the questionnaire were included in the study. Before participation, all students provided electronic informed consent after receiving a comprehensive explanation about study aims and procedures. The study was approved by the Ethics Committee of the University of Torino (protocol number 0574345, October 18, 2023) and was conducted in accordance with the ethical principles of the Declaration of Helsinki. The final analytical sample comprised 2,116 university students aged 18–39 years. No incentives or rewards were provided.

### Measures

#### Wellbeing

Mental wellbeing was assessed with the Warwick-Edinburgh Mental Wellbeing Scale (WEMWBS) (Tennant et al., 2007). The Italian version of the WEMWBS (Gremigni & Stewart-Brown, 2011) is a self-report questionnaire that uses a Likert scale from 1 (not at all) to 5 (always) to measure positive attitude towards life in the last two weeks through 12 items, such as ‘I’ve been feeling optimistic about the future’ and ‘I’ve been dealing with problems well’. This instrument evaluates mental wellbeing as a stable presence of positive states, and not just as the absence of negative symptoms or stress. The total scores of the 12 items were added together to create a total score, a higher score representing a higher state of mental wellbeing. The reliability of the Italian version was assessed by Gremigni and Stewart-Brown (2011) with a Cronbach’s Alpha between .83 and .86.

#### Stress

The original 14-item Perceived Stress Scale (PSS-14) was developed in 1983 by Cohen et al. (1983), but this first version was later revised and reduced to a 10-item version (Cohen, 1988). The Italian version of the PSS-10 (Mondo, Sechi, & Cabras, 2021) consists of 10 items and was used to measure the extent to which life was unpredictable, uncontrollable and overwhelming in recent weeks. It is based on a 5-point likert scale (0 = “never”,” 1 = “almost never”,” 2 = “sometimes”,” 3 = “quite often”,” 4 = “very often”). Examples of items are ‘how often have you felt that you were unable to control the important things in your life?’ and ‘how often have you been upset because of something that happened unexpectedly?’. After reversing the scores for the four positively formulated items (items 4, 5, 7 and 8), a total PSS10 score was calculated by adding all the items together. Higher scores indicate a higher perception of stress.

#### Anxiety and depression

Symptoms of anxiety and depression were assessed using the Hospital Anxiety and Depression Scale (HADS) (Zigmond & Snaith, 1983). The Italian version of the HADS (Costantini et al., 1999) is a self-report questionnaire consisting of 14 items rated on a 4-point Likert scale, designed to assess psychological distress over the past week. The instrument comprises two 7-item subscales measuring anxiety (HADS-A) and depression (HADS-D), with items such as “I feel tense or wound up” and “I feel as if I am slowed down.” Total scores for each subscale range from 0 to 21, with higher scores indicating greater symptom severity.

#### Lifestyle clustering

Lifestyle profiles were derived from six self-reported behavioural indicators: (i) sedentary behaviours, (ii) dietary habits, (iii) sleep habits, (iv) alcohol consumption, (v) sport participation, and (vi) smoking habits. Body mass index (BMI) was not included as an input variable in the final clustering procedure because it represents an anthropometric status indicator rather than a directly modifiable lifestyle behaviour and because preliminary analyses indicated that BMI had a negligible contribution to the original cluster structure. For the clustering procedure, all indicators were coded into categorical/ordinal variables as described below.

**Sedentary behaviour.** Sedentary behaviour was assessed by asking students to report the total time spent sitting or engaged in low-energy activities (e.g., studying at a desk, screen time) on a typical day. Participants reported the total amount of sedentary time, which was subsequently dichotomized after data collection using a threshold of 480 min/day (8 h/day), classifying participants as sedentary (≥480 min/day) or non-sedentary (<480 min/day).

**Dietary habits**. In this study, dietary habits were operationalized as adherence to the MD, assessed using the MediLite questionnaire (Sofi et al., 2014; Sofi et al., 2017). This validated instrument includes nine items evaluating the consumption of key food groups: daily intake of fruit, vegetables, cereals, meat and meat products, dairy products, alcohol, and olive oil, and weekly intake of legumes and fish. Each food group is classified into three intake categories based on cutoffs derived from the literature on Mediterranean dietary patterns and their associations with health outcomes. For typical MD food groups (fruit, vegetables, cereals, legumes, and fish), higher consumption corresponds to higher scores, whereas for non-typical food groups (meat and dairy products) lower consumption corresponds to higher scores. The sum of all items yields a total adherence score ranging from 0 to 18. For analytical purposes, the score was categorized into three ordered levels: low adherence (≤8 points), good adherence (9–11 points), and high adherence (≥12 points) (Cacciatore et al., 2023).

**Sleep habits**. Sleep habits were assessed using a single self-reported item on usual nocturnal sleep duration (in hours) on a typical night. In line with established sleep duration recommendations for adults and previous epidemiological research, responses were categorized into a three-level ordinal variable: short sleep duration (<6 hours), intermediate sleep duration (6–7 hours), and adequate sleep duration (>7 hours) (Hirshkowitz et al., 2015). These categories were used to approximate differences in habitual sleep patterns, considering that sleep durations below the recommended range are commonly associated with adverse health outcomes.

**Alcohol consumption**. Alcohol consumption was assessed by asking participants how many days they had consumed alcoholic beverages in the previous two months. Responses were recorded in ordered categories corresponding to the number of drinking days in the 60-day period (i.e., 0 days, 1–2 days, 3–5 days, 6–9 days, 10–19 days, 20–29 days).

**Sport participation**. Sport participation was assessed through an item asking about engagement in structured sport or exercise activities (e.g., team or individual sports, regular training sessions) during a typical week. Responses were coded into a binary indicator: Sportive (any sport participation) versus Non-sportive (no sport participation).

**Smoking habits (cigarettes and electronic cigarettes).** Smoking habits were derived from a yes/no smoking item with the reported number of cigarettes per day during the last two months. For clustering, responses were re-coded into a three-level categorical variable: non-smoker (0/day), light smoker (1–10/day), moderate smoker (11–20/day).

**Body Mass Index**. Body mass index (BMI, kg/m^2^) was calculated from self-reported weight and height and subsequently categorized according to standard WHO cut-points into four classes (WHO, 2000): underweight (<18.5 kg/m^2^), normal weight (18.5–24.9 kg/m^2^), overweight (25.0–29.9 kg/m^2^), and obesity (≥30.0 kg/m^2^).

### Statistical analysis

#### Lifestyle clustering

Lifestyle profiles were identified using a person-oriented clustering approach applied to six categorical lifestyle indicators. To reduce the risk of profiles driven by non-response, clustering was performed only on participants with sufficient observed information on the clustering indicators: individuals missing on ≥3 of the six categorical lifestyle indicators were excluded a priori. Pairwise dissimilarities between participants were computed using Gower’s coefficient, which accommodates variables measured on different scales, including categorical indicators (Gower, 1971). For two participants i and j, the overall dissimilarity was defined as d_ij_ = Σ_m_ w_ijm_d_ijm_ / Σ_m_ w_ijm_, where d_ijm_ is the variable-specific dissimilarity for variable m, and w_ijm_ indicates whether variable m is available for both participants. All six lifestyle indicators were treated as categorical variables. For categorical variables, the variable-specific dissimilarity was defined as d_ijm_ = 0 when participants i and j belonged to the same category, and d_ijm_ = 1 when they belonged to different categories. Clusters were derived using Partitioning Around Medoids (PAM), a k-medoids procedure that can be applied to a precomputed dissimilarity matrix and identifies observed representative cases as cluster medoids (Kaufman & Rousseeuw, 2009).

As the primary approach, missing values were represented as an explicit category for each indicator prior to distance computation. Sensitivity to missing-data handling was assessed for the selected solution by comparing this approach against an alternative specification in which missing values were retained as NA and handled within the Gower distance computation. Candidate solutions were explored for *k*=2–10. The final number of clusters was selected by integrating interpretability, cluster-size adequacy, silhouette width, bootstrap stability, Adjusted Rand Index (ARI), cell matching ratios (CMRs) analyses, and Gower-based homogeneity diagnostics. Internal validity was summarized using the average silhouette width; additional diagnostics included the proportion of negative silhouette widths, both overall and by cluster, and cluster-size adequacy. Structural changes across candidate solutions were examined using the Adjusted Rand Index (ARI) between adjacent solutions (*k* vs *k*+1). Cluster stability was further assessed via bootstrap resampling (B=50), comparing bootstrap-derived memberships with the corresponding full-sample solution using ARI.

Cluster homogeneity was additionally assessed using Gower-based compactness indices proposed for evaluating cluster-solution quality in person-oriented research (Vargha et al., 2016). These indices were summarized globally as cluster-size-weighted averages and examined across candidate solutions from *k* = 3 to *k* = 10 using scree plots. To complement ARI, CMRs were computed from pairwise contingency tables of cluster memberships and used as supplementary diagnostics to identify best-matching cluster pairs across adjacent solutions and in the missing-data sensitivity analysis (Vargha & Grezsa, 2024).

Associations between each lifestyle indicator and cluster membership were examined using chi-square tests with Cramér’s V as effect size. Monte Carlo p-values were obtained using chi-square tests with simulated p-values (B=5,000). BMI was retained as an external descriptive variable and analysed post hoc in relation to cluster membership. BMI categories were kept distinct as underweight, normal weight, overweight, and obesity. The association between BMI category and cluster membership was examined using a two-way frequency table and a chi-square test with Monte Carlo simulated p-values. Cramér’s V was used to quantify the magnitude of association.

As an additional sensitivity analysis addressing the ordinal nature of four lifestyle indicators, we repeated the PAMGower analysis using an ordinal-score specification in which ordered lifestyle categories were represented by numerical health scores before distance computation. Sedentary behaviour and sport participation were retained as binary 1/3 indicators, whereas dietary habits, sleep habits, alcohol consumption, and smoking habits were represented as ordered scores. The resulting *k*=4 partition was compared with the primary categorical-Gower solution using the ARI and CRMs.

All analyses were conducted in R (v4.5.2; R Foundation for Statistical Computing, Vienna, Austria) using RStudio (version 2026.01.0+392), with a fixed random seed set to 2025 for reproducibility. Clustering analyses used the *cluster, mclust*, and *rcompanion* packages.

#### Lifestyle health scores

After clustering, each lifestyle dimension was transformed into an ordinal health score ranging from 1 (least healthy) to 3 (most healthy) for descriptive profiling, graphical representation, and construction of the behavioural lifestyle index. Sedentary status was scored as non-sedentary=3 and sedentary=1. Diet and sleep were scored according to their ordered categories (diet: high=3, good=2, low=1; sleep: optimal=3, medium=2, poor=1). Alcohol frequency was scored as 0 days=3, 1–5 days=2, and ≥6 days=1. Sport participation was scored as sportive=3 and non-sportive=1. Smoking was scored using the three-level variable (nonsmoker=3; light smoker=2; moderate/heavy smoker=1). These numerical health scores were not used as metric input variables in the clustering procedure. Specifically, sedentary behaviour and sport participation were treated as binary categorical variables in the Gower dissimilarity computation. No intermediate score was assigned to these variables because the questionnaire did not include an empirically observed intermediate response level for sedentary status or sport participation.

#### ANCOVA

Between-cluster differences in psychological outcomes were assessed using analyses of covariance (ANCOVA) model using the following formula:

Psychological Outcome∼Cluster+Age+Gender

Age was modelled as a continuous covariate and mean-centred prior to analysis; gender was modelled as a binary covariate (Male/Female); participants reporting other gender categories were excluded from ANCOVA models due to very small cell sizes (i.e., 1.4%). Adjusted marginal means for each cluster were computed with 95% confidence intervals, and post-hoc pairwise comparisons were performed with Bonferroni adjustment. Effect sizes for the cluster term were quantified using partial eta-squared (*ηp^2^*). ANCOVA analyses were implemented in R (v4.5.2; R Foundation for Statistical Computing, Vienna, Austria) using RStudio (version 2026.01.0+392) using the packages *car, emmeans*, and *effectsize*.

### Results

#### Cluster solution selection and robustness

**Figure A1:**
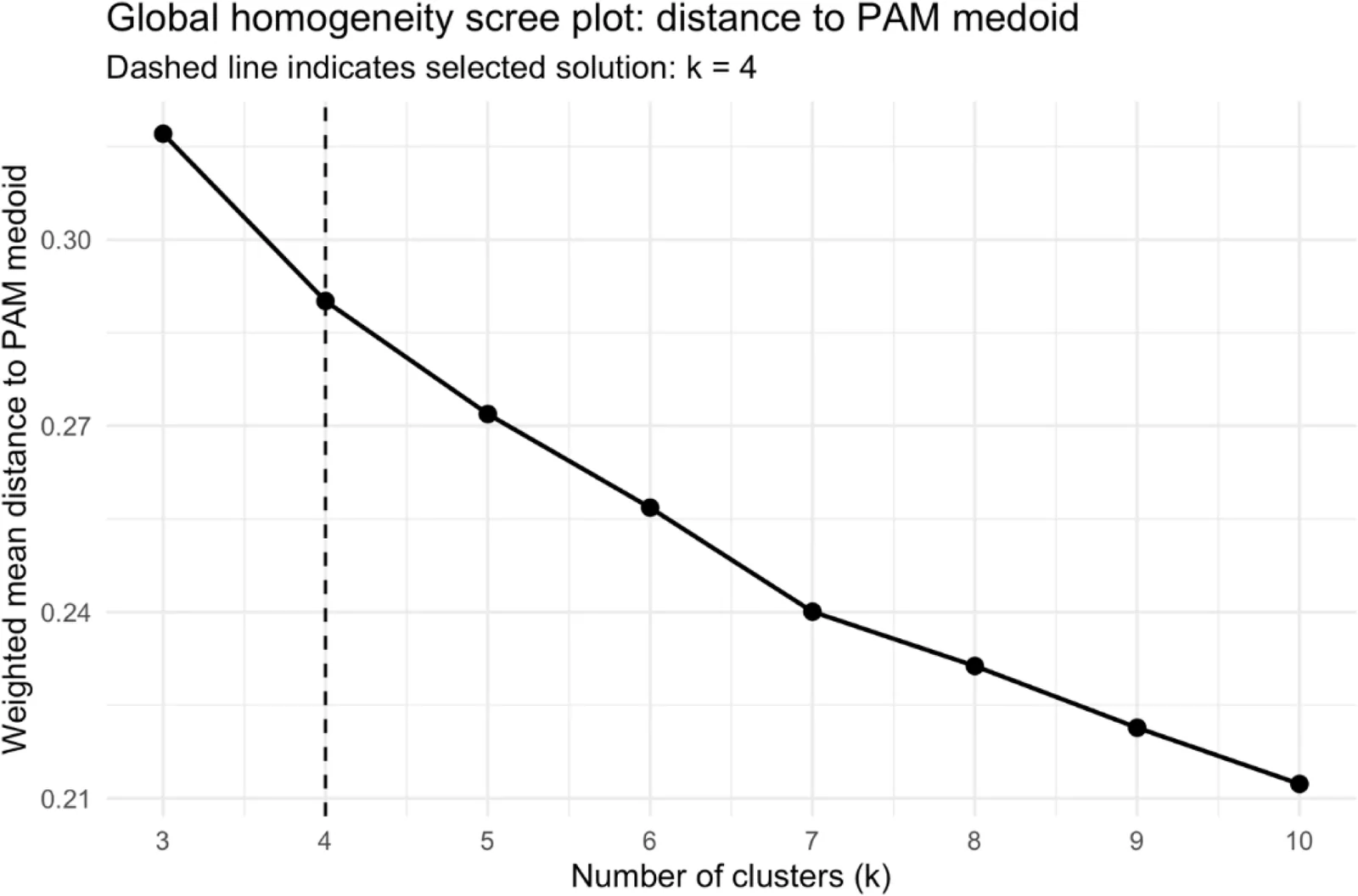
Scree plots of global homogeneity indices across k = 3 to k = 10.

Lifestyle profiles were derived in the clustering sample (≤ 2 missing values across the six lifestyle indicators; n=2,078). Gower-based homogeneity diagnostics supported the retained *k* = 4 solution, showing the largest improvement in compactness from *k* = 3 to *k* = 4 and lower compactness for Cluster 3 than for the other profiles. Detailed homogeneity diagnostics are reported in the Appendix, Table A1 and A2, and Figure A1. CMR analyses supported the interpretation of the selected *k* = 4 solution. In the missing-data sensitivity analysis, reciprocal best matches were identified across specifications, although correspondence was moderate. Further details are provided in the Appendix, Table A3. The average silhouette width was 0.166, and cluster-level mean silhouettes were positive. Bootstrap resampling (B = 50) indicated moderate stability for final *k* = 4 (ARI median=0.560; IQR 0.327–0.800). Therefore, *k* = 4 was selected as the most robust compromise between interpretability, cluster-size adequacy, internal validity, bootstrap stability, homogeneity diagnostics, CMR interpretation, and substantive usefulness across the candidate solutions. Associations between lifestyle indicators and cluster membership (Cramér’s *V*) suggested that sedentary status showed the strongest differentiation across profiles (Cramér’s *V*=0.789), followed by alcohol consumption (Cramér’s *V*=0.346), sport participation (Cramér’s *V*=0.322), sleep habits (Cramér’s *V*=0.321), with smaller effects for dietary habits (Cramér’s *V*=0.265) and smoking habits (Cramér’s *V*=0.078). Post-hoc missingness checks did not identify any cluster with a high average proportion of missing information across the six clustering indicators, supporting that the profiles were not driven by differential non-response after the ex-ante missingness filter. BMI was analysed post hoc as an external descriptive variable. The association between BMI category and cluster membership was statistically significant but negligible in magnitude (Cramér’s *V* = 0.064), indicating that BMI did not meaningfully differentiate the behavioural lifestyle profiles.

In the ordinal-score Gower sensitivity analysis, the *k* = 4 solution showed a higher average silhouette width than the primary categorical-Gower solution (0.256 vs. 0.166), but agreement between partitions was moderate (ARI = 0.292). Reciprocal best-matching clusters were identified for all four clusters, with CMRs ranging from 0.590 to 0.814. The ordinal-score solution was strongly driven by sedentary status (Cramér’s *V* = 0.986), whereas dietary habits showed only a negligible association with the ordinal-score clusters (Cramér’s *V* = 0.069), indicating that this alternative specification yielded a less multidimensional partition. Detailed results are reported in the Appendix, Table A4.

#### Cluster labels

Cluster sizes ranged from 365 to 604 participants, corresponding to 17.6% to 29.1% of the clustering sample. Cluster labels were assigned a posteriori based on the dominant category patterns across the six lifestyle indicators (sedentary behaviour, sport participation, diet, sleep, alcohol consumption frequency and smoking). Table 1 presents the percentage distribution of the six lifestyle indicators in relation to the 4 clusters, while the observed medoid profiles for the final k = 4 solution are reported in Table 2.

**Table 1:** Sample characteristics and behavioural variables by lifestyle profile (k = 4). Values are reported as mean (SD) for age and as n (%) for categorical variables. Percentages are computed within the column (overall sample or each cluster). For each categorical variable, “Missing” is treated as an explicit category and is reported as the last row.

	All (N=2,078)	Cluster 1 (n=604)	Cluster 2 (n=365)	Cluster 3 (n=534)	Cluster 4 (n=575)
Age, Mean (SD)	21.3 (3.3)	21.3 (3.2)	21.4 (3.3)	21.3 (3.2)	21.1 (3.3)
Gender, n (%)					
Male	715 (34.4)	191 (31.6)	120 (32.9)	188 (35.2)	216 (37.6)
Female	1336 (64.3)	402 (66.6)	241 (66.0)	338 (63.3)	355 (61.7)
Non-binary / another gender identity	27 (1.3)	11 (1.8)	4 (1.1)	8 (1.5)	4 (0.7)
Missing	-	-	-	-	-
Sedentary Behavior, n (%)					
Sedentary (≥480 min/day)	934 (44.9)	86 (14.2)	365 (100.0)	438 (82.0)	45 (7.8)
Non-sedentary (<480 min/day)	1144 (55.1)	518 (85.8)	-	96 (18.0)	530 (92.2)
Missing	-	-	-	-	-
Dietary Habits, n (%)					
Low adherence (≤8 points)	801 (38.5)	102 (16.9)	205 (56.2)	132 (24.7)	362 (63.0)
Good adherence (9–11 points)	911 (43.8)	404 (66.9)	98 (26.8)	312 (58.4)	97 (16.9)
High adherence (≥12 points)	360 (17.3)	96 (15.9)	61 (16.7)	89 (16.7)	114 (19.8)
Missing	6 (0.3)	2 (0.3)	1 (0.3)	1 (0.2)	2 (0.3)
Sleep habits, n (%)					
Short sleep duration (<6 hours)	155 (7.5)	38 (6.3)	33 (9.0)	30 (5.6)	54 (9.4)
Intermediate sleep duration (6–7 hours)	920 (44.3)	97 (16.1)	264 (72.3)	145 (27.2)	414 (72.0)
Adequate sleep duration (>7 hours)	995 (47.9)	468 (77.5)	68 (18.6)	355 (66.5)	104 (18.1)
Missing	8 (0.4)	1 (0.2)	-	4 (0.7)	3 (0.5)
Alcohol Consumption, n (%)					
0 days	1029 (49.5)	496 (82.1)	270 (74.0)	108 (20.2)	155 (27.0)
1–2 days	526 (25.3)	37 (6.1)	-	248 (46.4)	241 (41.9)
3–5 days	210 (10.1)	26 (4.3)	42 (11.5)	61 (11.4)	81 (14.1)
6–9 days	97 (4.7)	18 (3.0)	21 (5.8)	26 (4.9)	32 (5.6)
10–19 days	36 (1.7)	2 (0.3)	8 (2.2)	15 (2.8)	11 (1.9)
20–29 days	10 (0.5)	NA	1 (0.3)	5 (0.9)	4 (0.7)
Missing	170 (8.2)	25 (4.1)	23 (6.3)	71 (13.3)	51 (8.9)
Sport Participation, n (%)					
Non-Sportive	558 (26.9)	85 (14.1)	59 (16.2)	319 (59.7)	95 (16.5)
Sportive	1275 (61.4)	453 (75.0)	266 (72.9)	143 (26.8)	413 (71.8)
Missing	245 (11.8)	66 (10.9)	40 (11.0)	72 (13.5)	67 (11.7)
Smoking Habits, n (%)					
Non-smoker (0/day)	1510 (72.7)	463 (76.7)	292 (80.0)	370 (69.3)	385 (67.0)
Light (1–10/day)	336 (16.2)	74 (12.3)	36 (9.9)	104 (19.5)	122 (21.2)
Moderate (11–20/day)	44 (2.1)	13 (2.2)	6 (1.6)	8 (1.5)	17 (3.0)
Missing	188 (9.0)	54 (8.9)	31 (8.5)	52 (9.7)	51 (8.9)

**Table 2:** *Observed medoid profiles for the final PAM-Gower* k = 4 *solution*

Cluster	Cluster label	Sedentary behaviour	Dietary habits	Sleep habits	Alcohol consumption	Sport participation	Smoking habits
1	Active–Healthier lifestyle	Non-sedentary	Good adherence	Optimal sleep	0 days	Sportive	Non-smoker
2	Sedentary–Sporty (low diet/sleep)	Sedentary	Low adherence	Medium sleep	0 days	Sportive	Non-smoker
3	Sedentary–Non-sporty (higher drinking)	Sedentary	Good adherence	Optimal sleep	1–2 days	Non-sportive	Non-smoker
4	Active–Riskier lifestyle	Non-sedentary	Low adherence	Medium sleep	1–2 days	Sportive	Non-smoker

#### Cluster 1 (n=604; 29%) – Active–Healthier lifestyle

This profile was characterised by a predominantly nonsedentary pattern (85.8%) combined with high sport participation (75.0%). Diet and sleep showed comparatively favourable distributions, with 66.9% in the good adherence category and 77.5% reporting adequate sleep duration. Alcohol consumption was largely absent (82.1% reporting 0 days in the last two months). Smoking was mostly absent (76.7% non-smokers).

#### Cluster 2 (n=365; 17.6%) – Sedentary–Sporty (low diet/sleep)

This profile combined systematic sedentary behaviour (100%) with high sport participation (72.9%). Dietary habits were less favourable, with a majority classified as low diet adherence (56.2%) and a smaller proportion as good adherence (26.8%). Sleep was mainly in the medium category (72.3%), with fewer reporting adequate sleep duration (18.6%). Alcohol consumption was often absent (74.0% reporting 0 days), although a non-negligible fraction reported 3–5 days (11.5%). Smoking prevalence was low (9.9% light smokers).

#### Cluster 3 (n=534; 25.7%) – Sedentary–Non-sporty (higher drinking)

This profile was defined by high sedentary behaviour (82.0%), with 59.7% classified as non-sportive and only 26.8% as sportive. Diet quality was relatively favourable (58.4% good adherence), and sleep was predominantly adequate (66.5%). The defining feature was a more frequent alcoholconsumption pattern: only 20.2% reported 0 days, whereas 46.4% reported 1–2 days and 11.4% reported 3–5 days (with additional participants in higher-frequency categories). Smoking was slightly more prevalent than in Clusters 1–2 (19.5% light smokers).

#### Cluster 4 (n=575; 27.7%) – Active–Riskier lifestyle

This profile showed an overall active pattern (92.2% nonsedentary; 71.8% sportive) coupled with less favourable behavioural risk indicators. Diet quality was predominantly low (63% low adherence; 16.9% good adherence), and intermediate sleep duration (72.0%). Alcohol consumption was more frequent: 27% reported 0 days, whereas 41.9% reported 1–2 days and 14.1% reported 3–5 days (with smaller proportions in higher-frequency categories). Smoking was comparatively more common, with the lowest proportion of non-smokers (67.0%) and the highest proportion of light smokers (21.2%).

**Figure 1:**
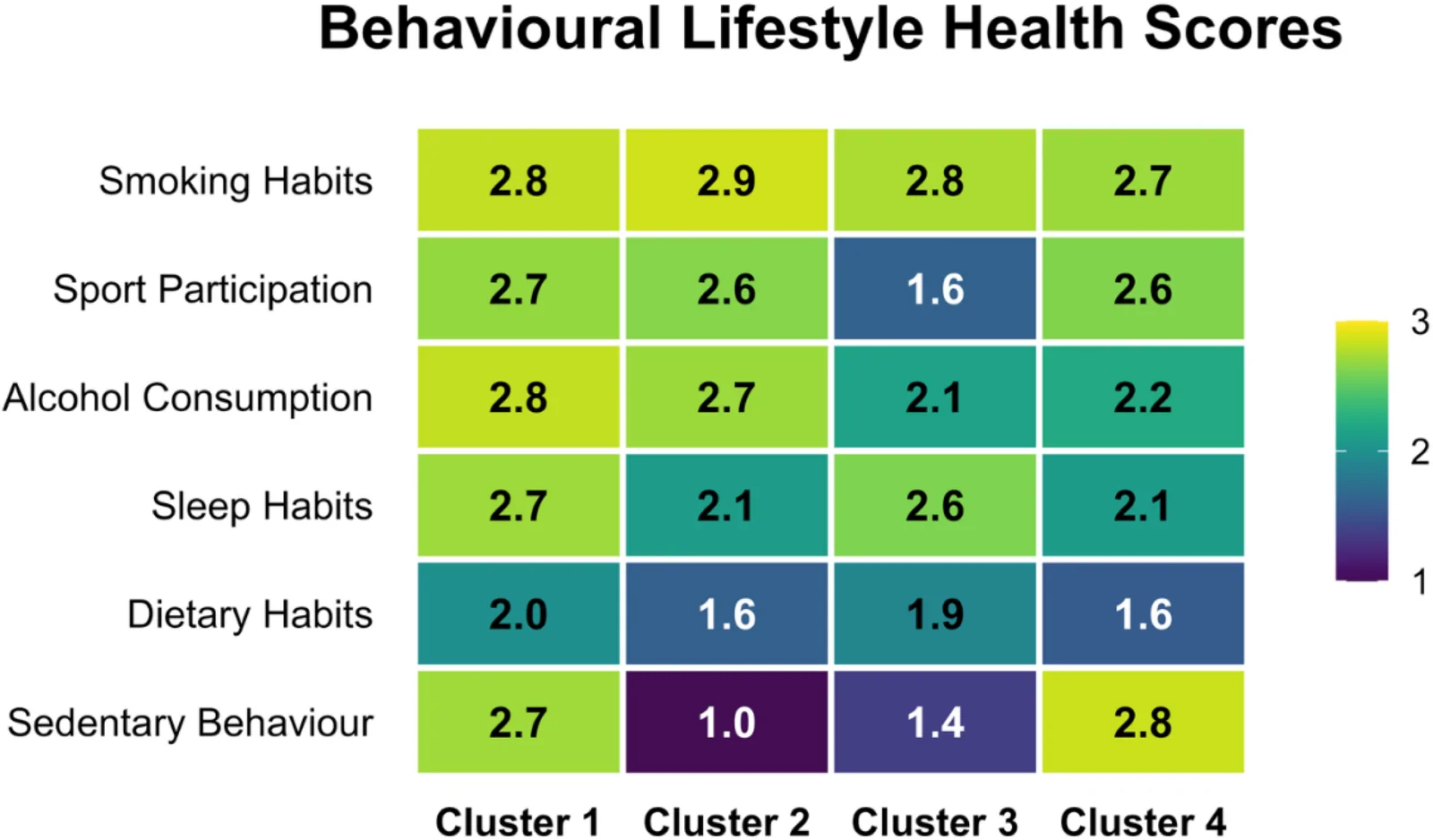
Heatmap reporting cluster-level means of the six behavioural lifestyle health scores used for post-clustering descriptive profiling. Scores range from 1 to 3, with higher values indicating healthier behaviours.

Figure 1 presents the lifestyle health scores of the lifestyle indicators in relation to the 4 clusters.

### ANCOVA results

After adjustment for age and gender, lifestyle profile membership was significantly associated with all psychological outcomes. Significant omnibus effects of lifestyle profile were observed for wellbeing (WEMWBS: *F*(3,2045) = 5.92, *p* < .001), perceived stress (PSS: *F*(3,2045) = 8.51, *p* < .001), anxiety (HADS Anxiety: *F*(3,2038) = 11.14, *p* < .001), and depression (HADS Depression: *F*(3,2045) = 7.30, *p* < .001). Effect sizes were small, with partial *η^2^* values ranging from 0.009 to 0.016. Bonferroni-adjusted post-hoc comparisons based on adjusted marginal means indicated that Cluster 1 Active–Healthier lifestyle showed more favourable psychological scores than the other profiles for most outcomes. For wellbeing, Cluster 1 reported higher WEMWBS scores than Cluster 2 Sedentary–Sporty (Δ = 1.35 points, *t*(2045) = 3.18, *p* = .009) and Cluster 3 Sedentary–Non-sporty (Δ = 1.46 points, *t*(2045) = 3.84, *p* < .001), whereas the difference between Cluster 1 and Cluster 4 Active–Riskier lifestyle was not significant after Bonferroni correction (*p* = .128). No other pairwise contrasts were significant for wellbeing. For perceived stress, Cluster 1 showed lower stress scores than Cluster 2 (Δ = 0.97 points, *t*(2045) = 2.90, *p* = .022), Cluster 3 (Δ = 1.40 points, *t*(2045) = 4.67, p < .001), and Cluster 4 (Δ = 1.14 points, t(2045) = 3.88, *p* < .001). Differences among Clusters 2, 3, and 4 were not significant. For anxiety, Cluster 1 reported lower HADS Anxiety scores than Cluster 2 (Δ = 0.99 points, *t*(2038) = 3.93, *p* < .001), Cluster 3 (Δ = 0.94 points, *t*(2038) = 4.17, *p* < .001), and Cluster 4 (Δ = 1.17 points, *t*(2038) = 5.30, *p* < .001). Differences among Clusters 2, 3, and 4 were not significant. Finally, for depression, Cluster 1 showed lower HADS Depression scores than Cluster 2 (Δ = 0.78 points, *t*(2045) = 3.92, *p* < .001), Cluster 3 (Δ = 0.52 points, *t*(2045) = 2.91, *p* = .022), and Cluster 4 (Δ = 0.69 points, *t*(2045) = 3.95, *p* < .001). Differences among Clusters 2, 3, and 4 were not significant. Table 3 reports the adjusted marginal means and 95% confidence intervals by lifestyle profile, together with the ANCOVA omnibus statistics.

**Table 3:** Adjusted marginal means and 95% confidence intervals by lifestyle profile and ANCOVA outcomes.

	Cluster 1 Active– Healthier lifestyle	Cluster2 Sedentary– Sporty	Cluster 3 Sedentary– Non-sporty	Cluster 4 Active– Riskier lifestyle	Omnibus ANCOVA	*ηp^2^* (magnitude)
WEMWBS	42.5 (42.0, 43.0)	41.1^*^ (40.5, 41.8)	41.0^*^ (40.5, 41.6)	41.6 (41.1, 42.1)	F(3,2045)=5.92, p<.001	0.009 (Small)
PSS	20.8 (20.4, 21.2)	21.7^*^ (21.2,22.3)	22.2^*^ (21.7, 22.6)	21.9^*^ (21.5, 22.3)	F(3, 2045)=8.51, p<.001	0.012 (Small)
HADS Anxiety	7.1 (6.8, 7.4)	8.1^*^ (7.7, 8.5)	8.1^*^ (7.7, 8.4)	8.3^*^ (8.0, 8.6)	F(3, 2038)=11.14, p<.001	0.016 (Small)
HADS Depression	6.4 (6.2, 6.7)	7.2^*^ (6.9, 7.5)	6.9^*^ (6.7, 7.2)	7.1^*^ (6.9, 7.3)	F(3, 2045)=7.30, p<.001	0.011 (Small)

Adjusted means are estimated marginal means from ANCOVA controlling for age and gender;

* , significant post-hoc difference from Cluster 1 Active–Healthier lifestyle. WEMWBS=Warwick-Edinburgh Mental Wellbeing Scale; PSS=Perceived Stress Scale; HADS=Hospital Anxiety and Depression Scale

## Discussion

This study explored the complex structure of health-related behaviours among university students, using a personoriented approach (Bergman & Magnusson, 1997) to address the limitations of traditional analyses that focus on individual risk factors (Zurc & Majerič, 2025).

A four-profile solution was retained as the most interpretable and methodologically adequate compromise across homogeneity diagnostics, cluster-size adequacy, internal validity, bootstrap stability, and substantive usefulness. Although cluster separation was modest (average silhouette width = 0.166), cluster-level mean silhouettes were positive and bootstrap resampling indicated moderate stability of the retained solution. The retained four-profile solution identified four empirically meaningful lifestyle configurations among university students, and profile membership was consistently associated with psychological outcomes. After adjusting for age and gender, lifestyle profiles differed across perceived wellbeing, perceived stress, anxiety, and depressive symptoms, with small but statistically reliable effects (partial *η^2^* ≈ 0.009–0.016). These effect sizes indicate that lifestyle profiles explain a limited but non-negligible portion of the variance in psychological outcomes. While they are not intended to predict individual mental health status, they provide meaningful information at the population level and may support the development of targeted health promotion strategies within university settings. In particular, Cluster 1 (Active–Healthier lifestyle) showed the most favourable psychological pattern across outcomes.

Consistent with recent evidence highlighting the multidimensional nature of lifestyles in young adulthood (Collins et al., 2023), the results identified four distinct profiles – Active–Healthier, Sedentary–Sporty, Sedentary–Non-sporty, and Active–Riskier – demonstrating that health-related behaviours rarely occur in isolation but rather cluster in complex and sometimes non-intuitive combinations. In other words, engagement in one healthy behaviour (e.g., sports participation) does not necessarily coincide with healthy patterns in other domains such as diet, sleep, or substance use. This contribution is especially relevant in the Italian university context, where studies that simultaneously integrate health behaviours and psychological wellbeing through a person-oriented approach are still limited. In line with the literature on emerging adulthood, the results confirm that the university period is a phase of particular vulnerability, during which increased decision-making autonomy and academic pressures can promote the adoption of suboptimal lifestyles, with the risk that these may persist into adulthood (Doak et al., 2023; Lonati et al., 2024; Wickham et al., 2020). Identifying distinct behavioural profiles thus enables a more realistic understanding of the complexity of students’ daily choices and provides a practical basis for targeted prevention interventions.

A particularly significant finding concerns the role of sedentary lifestyles, which emerged as the main distinguishing factor among the identified clusters. Among the six lifestyle indicators, sedentary status showed the strongest differentiation across profiles (Cramér’s *V* = 0.789), substantially exceeding the discriminative contribution of the remaining behavioural indicators. Although many students participate in sports, academic life involves long periods of physical inactivity—on average, about six hours a day are spent sitting, as is common for students attending classes and studying— which are not always offset by structured exercise (Lucini et al., 2024; Sansone et al., 2025). The distribution of sedentary behaviour across clusters highlights the heterogeneity of movement patterns among students and supports the need to distinguish sedentary time from sport participation. Notably, Cluster 2 and Cluster 3 were predominantly sedentary (100% and 82.0%, respectively), yet differed markedly in sport participation and other lifestyle domains, underscoring that prolonged sitting can coexist with both active and inactive leisure-time behaviours. Conversely, Cluster 1 and Cluster 4 were largely non-sedentary (85.8% and 92.2%), suggesting that profiles characterized by higher overall movement are not necessarily protected from other behavioural risks. This pattern aligns with the concept often described as the “active couch potato”, highlighting the conceptual distinction between engaging in structured physical activity and limiting sedentary time. Exercise may compensate only partially for prolonged sitting, which represents an independent behavioural dimension with distinct health implications (Bull et al., 2020; Tremblay et al., 2017). These findings reinforce the importance of addressing sedentary behaviour as a specific target of intervention, considering both physical/physiological and psychological needs, rather than assuming that participation in sport alone is sufficient to ensure a protective lifestyle.

The relationship between lifestyle and mental health is central to the findings. Cluster 1 (Active–Healthier) consistently reported the highest levels of wellbeing and the lowest levels of stress, anxiety, and depression, making it the most protective profile. For perceived stress, anxiety, and depressive symptoms, Cluster 1 differed significantly from all other profiles. For wellbeing, Cluster 1 reported significantly higher scores than Cluster 2 and Cluster 3, whereas the difference with Cluster 4 was not statistically significant after Bonferroni correction. Cluster 3 was characterised by high sedentary behaviour, lower sport participation, and more frequent alcohol consumption, and showed comparatively less favourable psychological scores. These results align with evidence indicating that quality sleep and physical activity level are among the strongest predictors of psychological wellbeing and flourishing in young adulthood (Altun et al., 2012; Wickham et al., 2020). One possible interpretation is the presence of a threshold-like pattern, whereby maintaining an overall favourable lifestyle profile may be psychologically protective, whereas variation among suboptimal profiles may not be sufficient to produce clearly differentiated levels of distress. Although the cross-sectional design of the study does not allow for causal inferences, the observed associations may plausibly be reciprocal. Academic stress can, in certain cases, encourage dysfunctional coping strategies, such as alcohol consumption or increased sedentary behaviour, while these behaviours may contribute to worsening anxiety and depressive symptoms, creating a vicious cycle that undermines psychological wellbeing and, over time, cardiometabolic health (Bourke, Brown & Kwan, 2025; Collins et al., 2023; Simões de Almeida et al., 2025).

Another interesting element is Cluster 4 (Active–Riskier), which consists of physically active students who are more likely to engage in risky behaviours such as smoking and alcohol consumption. This profile, already documented in previous studies, suggests that some young people may view physical exercise as a compensatory strategy for unhealthy habits, or that sports contexts themselves facilitate social consumption practices (Sansone et al., 2025; Swelam et al., 2022). Furthermore, nutritional quality was found to be suboptimal across all identified profiles, confirming widespread adherence to Western-style eating patterns. In particular, Cluster 4 showed the highest proportion of low dietary adherence (63.0%) despite high levels of sport participation and non-sedentary behaviour. This finding further illustrates that favourable behaviours in one domain do not necessarily extend to other health-related domains.

Interestingly, BMI category showed only negligible association with cluster membership (Cramér’s *V* = 0.064). This suggests that the behavioural lifestyle profiles captured patterns of everyday health-related practices that were largely independent of body-weight status. In this university population, behavioural heterogeneity therefore appeared more informative than anthropometric status alone for describing lifestyle variation.

Overall, the distinction among the four clusters underscores the importance of moving beyond generic health promotion approaches in universities in favour of tailored strategies that address the multidimensional nature of behavioural profiles. Importantly, the identified profiles are not only descriptive but also operational, as each cluster points to different behavioural leverage points for intervention. For example, students in Cluster 3 may benefit from initiatives aimed at reducing sedentary behaviour and improving stress management, whereas students in Cluster 4 may require interventions focused on food literacy and awareness of the risks associated with substance use. More broadly, the results indicate that effective campus interventions should not be limited to promoting sport participation alone but should simultaneously address sedentary time, dietary quality, sleep habits, and substance use. Universities, as true public health hubs, play a crucial role in shaping the wellbeing trajectories of young adults by offering services tailored to the needs of different subgroups (de Lima et al., 2025; Doak et al., 2023). Since young adults often perceive themselves as healthy based on clinical parameters that are still within normal ranges, communication strategies are more effective when they emphasize the immediate benefits of healthy choices – such as improved vitality, concentration, and quality of life – rather than the prevention of future risks. In this context, improving sleep hygiene emerges as a fundamental pillar (Oftedal et al., 2024), given its close association with mental wellbeing and daily functioning during the academic years (Vestergaard et al., 2024).

This study has some limitations that should be considered. Although the sample included individuals aged 18 to 39, the majority of participants were concentrated among the younger ages (21 ± 3 years). Consequently, while all participants were included in the analyses, the adjustment for age primarily reflects the effect within the younger age range and may not fully account for potential confounding in the older age groups. BMI was available only as a descriptive anthropometric indicator and was not used to derive the behavioural profiles. Although BMI is widely used in epidemiological research, it does not capture body composition and cannot distinguish fat mass from fat-free mass. This study did not consider whether participants were working in addition to studying. Employment status could influence the considered behavioural and anthropometric indicators, as well as the psychological outcomes, and its omission may represent a residual confounding factor. A further limitation is that sedentary behaviour and sport participation were available only as binary indicators. Although this coding was handled categorically in the Gower dissimilarity matrix, it may have reduced behavioural granularity. Future studies should use more detailed measures capturing frequency, duration, intensity, and domain-specific patterns of sedentary behaviour and physical activity/sport participation. Finally, although the primary clustering solution treated all lifestyle indicators as categorical variables in the Gower dissimilarity matrix, an ordinal-score sensitivity analysis showed that individuallevel cluster assignments were moderately sensitive to the treatment of ordinal information. The ordinal-score solution showed higher compactness but was strongly dominated by sedentary status, suggesting a trade-off between preserving ordinal spacing and retaining multidimensional behavioural profiles.

In conclusion, the present findings indicate that behavioural lifestyles among university students are multidimensional and not reducible to single health behaviours. The identification of a more favourable lifestyle profile, together with three distinct but psychologically comparable suboptimal profiles, highlights the value of person-oriented approaches for describing behavioural heterogeneity in university populations. Since the university period represents a unique window of opportunity for primary prevention, investing in policies that transform the campus into an environment that facilitates healthy choices is essential to promote long-term wellbeing.

## Data Availability

The data presented in this study are available on request from the corresponding author due to being part of the Wellness for Students (W4S) project.

## Action editor

Lars-Gunnar Lundh served as action editor for this article.

## Open access

This article is distributed under the terms of the Creative Commons Attribution 4.0 International License (http://creativecommons.org/licenses/by/4.0/), which permits unrestricted use, distribution, and reproduction in any medium, provided you give appropriate credit to the original author(s) and the source, provide a link to the Creative Commons license, and indicate if changes were made.

## Appendix

**Table A1:** Global homogeneity diagnostics across PAM-Gower candidate solutions from k = 3 to k = 10.

*k*	Weighted mean within-cluster Gower distance	Weighted mean distance to PAM medoid
3	0.467	0.317
4	0.434	0.290
5	0.413	0.272
6	0.393	0.257
7	0.376	0.240
8	0.362	0.231
9	0.345	0.221
10	0.332	0.212

**Table A2:** Cluster-level and global homogeneity diagnostics for the final PAM-Gower k = 4 solution.

Cluster	n	%	Average within-cluster Gower distance	Average distance to PAM medoid
1	604	29.1	0.371	0.227
2	365	17.6	0.372	0.241
3	534	25.7	0.514	0.363
4	575	27.7	0.464	0.320
**Global weighted mean**	2,078	100.0	**0.434**	**0.290**

**Table A3:** Cell matching ratios for reciprocal best-matching cluster pairs across PAM-Gower solutions and missing-data specifications.

Comparison	Cluster in solution A	Cluster in solution B	CMR	Interpretation
*k* = 3 vs *k* = 4	C1	C1	1.000	Preserved
*k* = 3 vs *k* = 4	C2	C2	1.000	Preserved
*k* = 3 vs *k* = 4	C3	C3	1.000	Preserved
*k* = 4 vs *k* = 5	C1	C1	0.927	Largely preserved
*k* = 4 vs *k* = 5	C2	C5	0.904	Largely preserved
*k* = 4 vs *k* = 5	C3	C3	1.000	Preserved
*k* = 4 vs *k* = 5	C4	C4	0.581	Partly fragmented
Missing-level vs NA-Gower, *k* = 4	C1	C4	0.669	Moderate correspondence
Missing-level vs NA-Gower, *k* = 4	C2	C2	0.548	Moderate correspondence
Missing-level vs NA-Gower, *k* = 4	C3	C3	0.457	Moderate-to-weak correspondence
Missing-level vs NA-Gower, *k* = 4	C4	C1	0.763	Moderate-to-good correspondence

**Table A4:** Ordinal-score Gower sensitivity analysis comparing the primary categorical-Gower solution with the ordinal-score Gower solution (k=4, n=2,078).

Average silhouette	Categorical-solution	Ordinal-solution

	0.166	0.256
ARI between categorical- and ordinal-solutions	0.292	
Reciprocal best-matching clusters	4/4	
Reciprocal CMR range	0.590-0.814	
Ordinal-score cluster sizes	551, 509, 440, 578	
**Cramer’s V with ordinal-clusters**		
Sedentary status	0.986	
Sleep habits	0.459	
Sport participation	0.227	
Alcohol consumption	0.159	
Smoking habits	0.145	
Dietary habits	0.069	
**Reciprocal best-matching clusters**		
Categorical C1 -> Ordinal C1	Overlap = 325; CMR = 0.590	
Categorical C2 -> Ordinal C2	Overlap = 297; CMR = 0.814	
Categorical C3 -> Ordinal C3	Overlap = 294; CMR = 0.668	
Categorical C4 -> Ordinal C4	Overlap = 370; CMR = 0.643	

*Note*: CMR = Cell Matching Ratio; C1 = Cluster 1; C2 = Cluster 2; C3 = Cluster 3; C4 = Cluster 4.
